# Outer Segment Formation of Transplanted Photoreceptor Precursor Cells

**DOI:** 10.1371/journal.pone.0046305

**Published:** 2012-09-28

**Authors:** Dominic Eberle, Thomas Kurth, Tiago Santos-Ferreira, John Wilson, Denis Corbeil, Marius Ader

**Affiliations:** 1 DFG-Center for Regenerative Therapies Dresden (CRTD), Cluster of Excellence/TU-Dresden, Dresden, Germany; 2 Biotechnology Center, TU-Dresden, Dresden, Germany; 3 Verna and Marrs McLean Department of Biochemistry and Molecular Biology, Baylor College of Medicine, Houston, Texas, United States of America; Center of Ophtalmology, Germany

## Abstract

Transplantation of photoreceptor precursor cells (PPCs) into the retina represents a promising treatment for cell replacement in blinding diseases characterized by photoreceptor loss. In preclinical studies, we and others demonstrated that grafted PPCs integrate into the host outer nuclear layer (ONL) and develop into mature photoreceptors. However, a key feature of light detecting photoreceptors, the outer segment (OS) with natively aligned disc membrane staples, has not been studied in detail following transplantation. Therefore, we used as donor cells PPCs isolated from neonatal double transgenic reporter mice in which OSs are selectively labeled by green fluorescent protein while cell bodies are highlighted by red fluorescent protein. PPCs were enriched using CD73-based magnetic associated cell sorting and subsequently transplanted into either adult wild-type or a model of autosomal-dominant retinal degeneration mice. Three weeks post-transplantation, donor photoreceptors were identified based on fluorescent-reporter expression and OS formation was monitored at light and electron microscopy levels. Donor cells that properly integrated into the host wild-type retina developed OSs with the formation of a connecting cilium and well-aligned disc membrane staples similar to the surrounding native cells of the host. Surprisingly, the majority of not-integrated PPCs that remained in the sub-retinal space also generated native-like OSs in wild-type mice and those affected by retinal degeneration. Moreover, they showed an improved photoreceptor maturation and OS formation by comparison to donor cells located on the vitreous side suggesting that environmental cues influence the PPC differentiation and maturation. We conclude that transplanted PPCs, whether integrated or not into the host ONL, are able to generate the cellular structure for effective light detection, a phenomenon observed in wild-type as well as in degenerated retinas. Given that patients suffering from retinitis pigmentosa lose almost all photoreceptors, our findings are of utmost importance for the development of cell-based therapies.

## Introduction

Retinitis pigmentosa (RP), a collective term for a group of inherited retinal eye diseases, represents, together with age-related macula degeneration (AMD), one of the main causes for visual impairment and blindness in industrialized countries. The dominant reason for vision loss is, in both cases, the irreversible loss of photoreceptor cells located in the outer nuclear layer (ONL) of the retina. To date, no effective treatment is available to preserve or regain visual function in affected patients. Transplantation of photoreceptor precursor cells (PPCs) into the retina represents a recent promising treatment for photoreceptor replacement in blinding diseases characterized by photoreceptor cell loss.

By following experiments initiated already two decades ago by Gouras and colleagues [1]–[3], several recent studies are developing cell replacement strategies for degenerated photoreceptor cells using diverse cell populations including pluripotent stem cells [4]–[7] or cells derived from the retina [8]–[13]. In preclinical studies it was demonstrated that donor PPCs isolated directly from the neonatal mouse retina at postnatal day (PN) 3–5 have the highest potential to develop into mature photoreceptors [10], [11], [14], [15], which form axonal terminals and inner (IS) and outer (OS) segments [12] following grafting into the retina of adult hosts. While a properly developed OS with well-aligned disc membrane staples is crucial for light detection and conversion into an electric signal, an axonal terminal that connects to the respective bipolar cell is indispensable for transmitting the electric signal to the host neural circuitry. Functional analyses, such as electroretinogram (ERG) recordings, pupillary reflexes, optokinetic tracking or visual Morris water maze, have been described after transplantation of PPCs into murine models of retinal degeneration (RD) suggesting improvements in visual function [4]–[6], [10], [13]. Nevertheless, these studies still lack the direct morphological evidence for proper OS formation.

Pioneer studies by Gouras et al. [1]–[3] and Bartsch et al. [11] suggesting OS formation of transplanted photoreceptors were impeded due to the lack of optimal labeling methods of donor OSs. Similarly, all other studies on photoreceptor transplantation failed as well to demonstrate formation of OS due to the absence of fluorescent reporter proteins in the OS of transplanted PPCs [10], [11], [13]–[15]. Here, we took advantage of a recently generated transgenic reporter mouse line in which enhanced green fluorescent protein (EGFP) is fused to human rhodopsin protein, the main photopigment in rod photoreceptors [16], to investigate these issues. Rhodopsin is exclusively located to the OS of mature rod photoreceptors allowing detailed ultra-structural analysis of their formation and integrity upon the transplantation of PPCs into adult mouse retinas. Using such model organisms combined with the isolation of PPCs based on CD73 as a cell surface marker we successfully demonstrated that transplanted PPCs are able to form a native OS including the formation of a connecting cilium and well-aligned disc membrane staples, the latter structure being an indispensable morphological prerequisite for proper light detection. Remarkably, the OS formation was not restricted to transplanted cells correctly integrated into the ONL but was also observed when photoreceptors remained in the sub-retinal space of the host.

## Results

### Visualization of outer segments using fluorescence light and electron microscopy

Correlative light and electron microscopy (CLEM) combines fluorescent light microscopic (FLM) labeling with electron microscopy (EM), enabling studies of bulky tissue samples from low to sub-cellular high resolution [17]–[19]. We took advantage of the CLEM flexibility to localize the rare events of PPC integration, and to study the formation of OS at an ultra-structural resolution.

**Figure 1:**
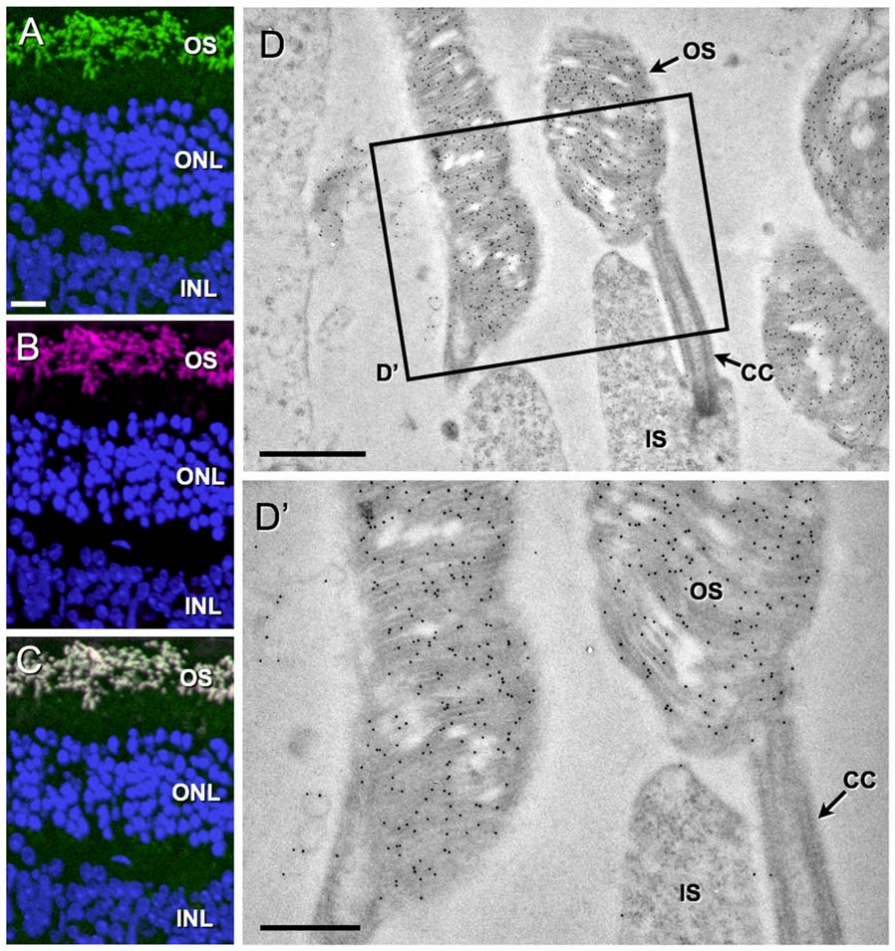
Ultra-structural identification of outer segments from rhoEGFP transgenic mice. The 200-nm thin K4M-resin sections of a retina isolated from an adult rhoEGFP mouse were immuno-stained with anti-EGFP antibodies followed by goat anti-rabbit Alexa555 (A–C). RhoEGFP fluorescence (A, green) is restricted to the outer segments (OS) of photoreceptors and completely overlaps with the immuno-fluorescent signal (B, magenta; C is the merged image of A and B). Outer (ONL) and inner (INL) nuclear layers are highlighted by DAPI staining (blue). For ultra-structural analysis, resin sections from a rhoEGFP retina were immuno-stained with anti-EGFP antibodies, followed by protein A 10-nm gold (D; D′ is a high power image of the boxed region in D). Note the specific immuno-labeling of OSs containing the typical disc membrane staples characteristic for mature photoreceptors. CC, connecting cilium; IS, inner segment. Scale bars: A–C: 20 µm, D: 1 µm, D′: 500 nm.

To demonstrate the feasibility of immuno-CLEM on retina samples as a proof of principle, adult eyes from a rhoEGFP mouse were isolated, embedded in Lowicryl K4M resin, and ultra-thin sections were immuno-labeled using anti-GFP antibodies (Fig. 1). The rhoEGFP transgenic animals was characterized by the expression of EGFP fused to human rhodopsin [16], which is exclusively located in the photoreceptor OS (Fig. 1A). As expected, a strong overlapping signal of rhoEGFP per se and the immuno-labeling (Fig. 1B) was observed exclusively within OSs (Fig. 1C). At transmission electron microscope (TEM) level, sections derived from the same block preparation labeled with anti-GFP antibodies followed by protein A 10-nm gold revealed that immuno-labeling is specific for photoreceptor OSs demonstrating that these light detecting structures can be visualized at an ultra-structural resolution based on a targeted EGFP expression (Fig. 1D, D′).

### Transplanted photoreceptor precursor cells integrate into wild-type host retinas and form native outer segments with well-aligned disc membrane staples

To demonstrate whether PPCs transplanted into a wild-type (wt) retina develop proper OSs, we used a dual strategy based on one hand on their paramagnetic immuno-isolation by means of CD73 as a selective cell surface marker [12], and on the other hand on double-(actinDsRed, rhoEGFP) transgenic reporter animals, which allow to visualize the transplanted cells in the host wt retina. Indeed, the actin-driven DsRed expression highlights the entire cell body of transplanted PPCs with exception of the OS (for details see Materials and Methods) that is labeled by EGFP (i.e. rhoEGFP, see above). Donor PPCs were isolated from postnatal day (PN) 4 animals for all experiments in this study.

**Figure 2:**
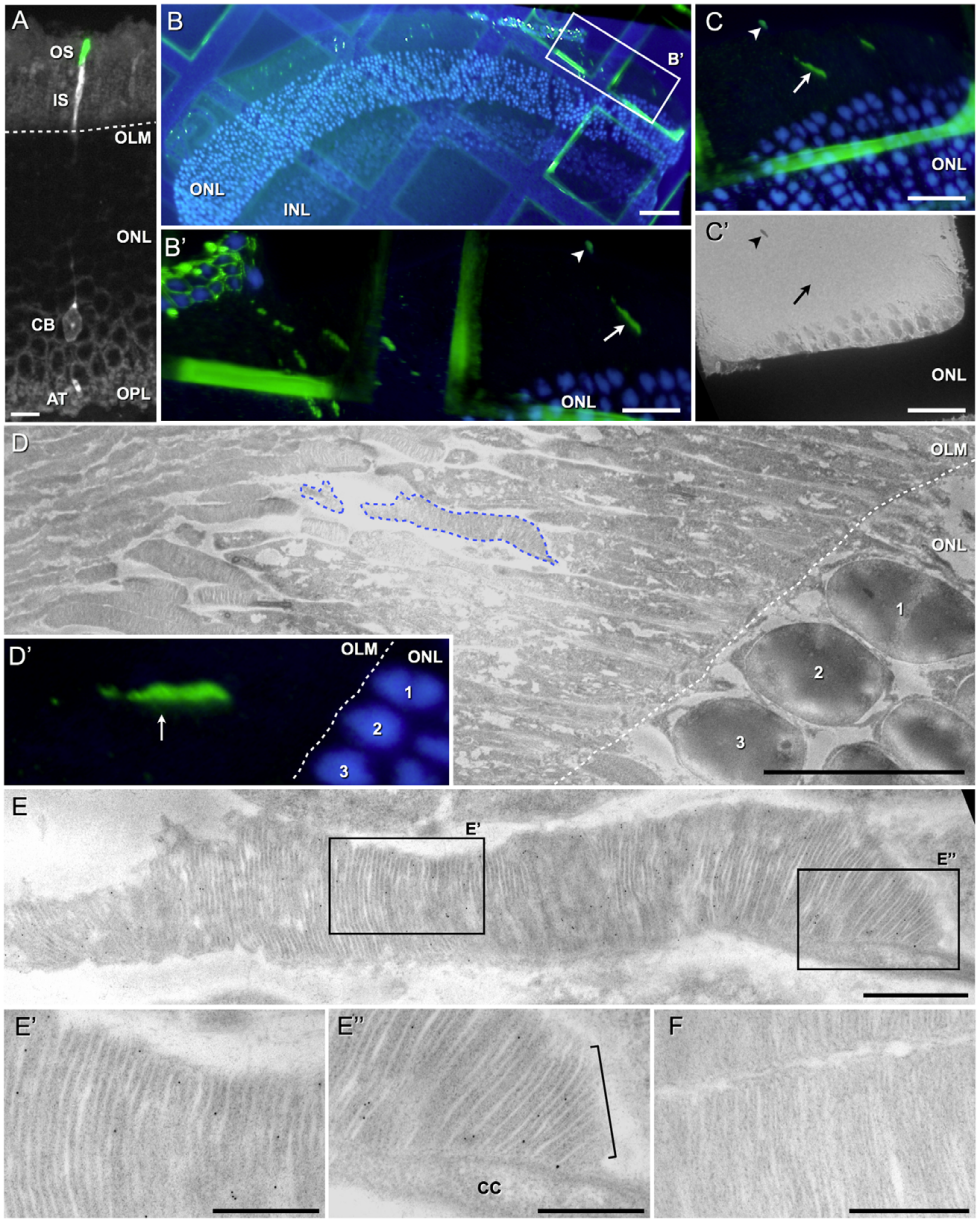
Integrated donor rhoEGFP–positive photoreceptors generate outer segments in wild-type host retinas. Upon transplantation into an adult wild-type mouse (A), CD73-enriched photoreceptor precursor cells (PPC) isolated from double-transgenic (rhoEGFP, actinDsRed) reporter animals integrated properly into the host retina and developed the typical morphology of mature photoreceptors, including a round cell body (CB, white) located in the outer nuclear layer (ONL), an axonal terminal (AT) in the outer plexiform layer (OPL) as well as an inner (IS, white) and outer (OS, green) segment located above the outer limiting membrane (OLM, dashed line). On a representative correlative light electron microscopy section (B; B′ is a higher power view of the boxed area in B) rhoEGFP–positive elongated structures could be observed (B′, arrow). Cell nuclei are stained with DAPI (blue). Note that not every green fluorescent signal in the area of host OSs indicated the presence of biological structures since unspecific signals (B′, C and C′, arrowhead) could mimic the typical ones of a rhoEGFP–positive outer segment (C, arrow), which was not observable in low-magnification transmission electron microscopy (TEM) in contrast to the contamination (C′, arrow and arrowhead, respectively). RhoEGFP–positive OSs derived from integrated, transplanted PPCs showed the physiological elongated morphology as observed by TEM (D, blue dashed line; E) and the corresponding fluorescent light microscopy image (D′, arrow; as reference points three nuclei were numbered). The boxed areas in E are shown at higher magnification in the respective panels E′ and E″. Note that in addition to the formation of a connecting cilium (E″, CC) the donor-derived OSs displayed growing membrane evaginations at their bases (E″, bracket) and typical densely packed disc membrane staples (E′, E″) similar to endogenous OSs (F). Scale bars: A: 10 µm, B: 50 µm, B′–C′: 20 µm, D: 10 µm, E: 1 µm, E′–F: 500 nm.

Three weeks post-transplantation, a sub-fraction of transplanted PPCs successfully integrated into the ONL of wt mice with an integration rate similar to our previous investigations, i.e. about 2000±1000 cells/retina [12]. Donor cells showed DsRed-expression in the whole cell body including the axonal terminal connection within the outer plexiform layer (OPL) (Fig. 2A, white), while rhoEGFP-expression was detectable exclusively in elongated, tubular structures of approximately 5–15 µm length and 1 µm width apical to the rest of the cell body (Fig. 2A, green). Since the latter structure has the typical shape and localization of a photoreceptor OS, it is highly probable that transplanted PPCs develop an OS, too. CLEM analysis of single ultra-thin sections mounted to finder grids (Fig. 2B) revealed rhoEGFP–positive elongated tubular structures (Fig. 2B′: boxed area in B; Fig. 2C, C′, arrow).

At high-resolution level, these fluorescent structures (e.g. see Fig. 2D′, arrow) turned out to be morphologically normal OSs (Fig. 2D, TEM-micrograph corresponding to FLM image in D′). Thus, OSs of the transplanted cells displayed well-aligned disc membranes growing out from a connecting cilium (Fig. 2E–E″). Although the overall membranous organization of the vast majority (20 out of 21) of analyzed transplanted rhoEGFP–positive photoreceptor OSs was indistinguishable to that of native photoreceptor cells found in the host retina (Fig. 2F), we observed in few cases donor cells harboring slight disturbances of the alignment of disc membranes within OSs (data not shown). Such phenomena might be the result of the sample preparation or might represent individual cellular variances, since they were also observed in rare cases in host photoreceptor OSs.

Technically, it is important to point out that a careful observation and interpretation of fluorescent OSs are crucial since certain unidentified auto-fluorescent structures in the area of host OSs (Fig. 2B′, C, arrowhead), might correspond to false–positive signals as detected by TEM (Fig. 2C′). Taken together, we demonstrated technically a powerful method to analyze the ultra-structure of the OSs of transplanted PPCs into host retinas, and revealed from a cell biological point of view that PPCs integrated into the host ONL are able to develop morphologically normal OSs.

### Non-integrated cells in the wild-type sub-retinal space develop an outer segment

Several studies suggest that transplanted photoreceptors form OSs when integrating into the ONL of experimental animals [1], [2], [10]–[13]. However, patients with severe RD are characterized by an almost absence of photoreceptor cells and are thus missing a proper ONL structure that would allow integration of donor cells. Indeed, the integration rate of transplanted PPCs strongly decreases in mouse models of RD characterized by a severe loss of the ONL ([20], [21] and see below) and non-integrated donor photoreceptors fail to form the typical elongated morphology of mature wt photoreceptors. Also, following transplantation into a normal environment, the majority of grafted photoreceptors remains in the sub-retinal space and does not develop photoreceptor morphology [11].

**Figure 3:**
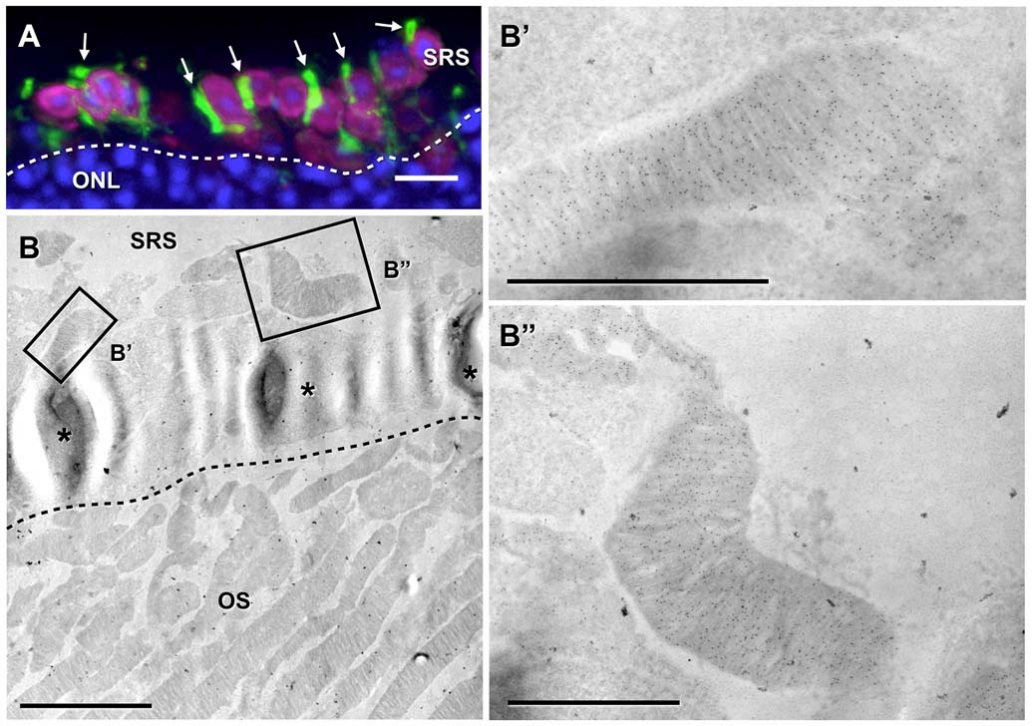
Transplanted photoreceptors remaining in the sub-retinal space generate outer segments. Upon transplantation into an adult wild-type mouse (A), many CD73-enriched photoreceptor precursor cells (PPC) isolated from double-transgenic (rhoEGFP, actinDsRed) reporter animals remain in the sub-retinal space (SRS) at the top of the host outer nuclear layer (ONL, dashed line) and develop outer segment (OS)-like structures that are labeled with rhoEGFP (green, arrows). The cell body of transplanted cells is observed by DsRed-expression (magenta) whereas cell nuclei are stained with DAPI (blue). An electron microscopy overview (B) of transplanted PPCs found in the SRS demonstrates that several of them (asterisks) lay above the host photoreceptor OSs, which are delimitated by a dashed line. Magnifications of the boxed areas in panel B reveal that the non-integrated PPCs developed as well organized disc membrane staples, which are positive for rhoEGFP (B′, B″, black dots). Scale bars: A: 10 µm, B: 5 µm, B′ and B″: 2 µm.

Using CD73-enriched donor cells isolated from double-(actinDsRed, rhoEGFP) transgenic reporter mice we observed that transplanted cells located in the sub-retinal space of wt hosts do not develop the elongated morphology of integrated photoreceptors as seen in Figure 2A. In contrast, we observed round DsRed–positive cell bodies with adjacent longitudinal, EGFP–positive, OS-like structures (Fig. 3A, arrows). Ultra-structural analysis revealed that EGFP–positive extensions were native-like OSs containing well-aligned disc membrane staples (Fig. 3B–B″) in the majority of studied OSs (35 out of 40). Therefore, we conclude that donor photoreceptors not only form native-like OSs when correctly integrating into the ONL of the host but also when remaining in the sub-retinal space.

### Transplanted photoreceptor precursor cells do not integrate in P347S host retinas but form outer segments in the sub-retinal space

In the previous sections we have shown that PPCs have the potential to form OSs when integrated into the ONL or remaining in the sub-retinal space of wt hosts. Based on these results, we further investigated whether a similar phenomenon might occur in severely degenerated retinas. P347S transgenic mice represent a valuable model for autosomal-dominant RD, and heterozygous animals loose the majority of photoreceptors within 12 weeks ([22], our unpublished observations).

**Figure 4:**
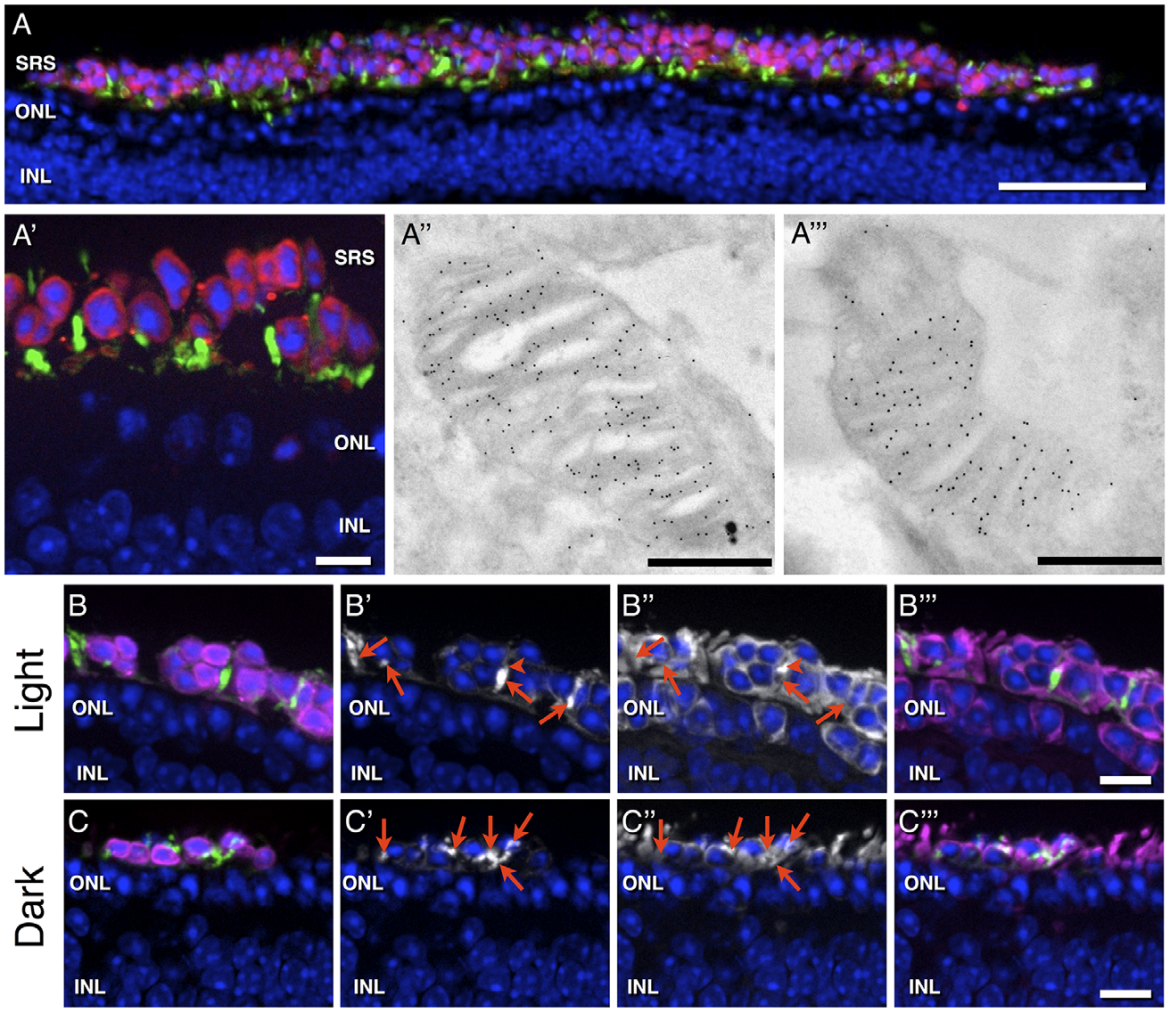
Transplantation of rhoEGFP–positive photoreceptors into the degenerated retina of heterozygous P347S mice. Low and high magnification views (A, A′) of a degenerated retina from a heterozygous P347S mouse, 3 weeks post-transplantation of CD73-enriched PPCs isolated from double-transgenic (rhoEGFP, actinDsRed) reporter animals. Cell nuclei are stained with DAPI (blue). Note, that transplanted cells (cell body, magenta; outer segment, green) are found in a sheet-like structure in the sub-retinal space (SRS), i.e. above the host outer nuclear layer (ONL), which is reduced to only 1–2 cell rows. Electron microscopy analyses of rhoEGFP–positive structures reveal the formation of ultra-structurally normal OSs containing morphologically native disc membrane staples (A″, A′″). In light adapted animals (B–B′″), transducin (B″, white and B′″, magenta) is expressed in OSs of transplanted, sub-retinal PPCs at low levels (B′ and B″, arrows), whereas high levels of transducin could be detected in IS-like structures next to rhoEGFP-positive OSs (B′ and B″, arrowhead). In contrast, dark adapted animals show significantly higher levels of transducin in rhoEGFP-positive OSs (C′ and C″, arrows), indicated also by white overlay staining in C′″. This illustrates, that sub-retinal PPCs show the native translocation of transducin under different light conditions, which is a prerequisite for functionality. INL, inner nuclear layer. Scale bars: A: 50 µm, A′: 10 µm, A″, A′″: 500 nm, B–B′″ and C–C′″: 10 µm.

Following sub-retinal transplantation of CD73-enriched PPCs derived from double-(actinDsRed, rhoEGFP) transgenic mice into heterozygous P347S mouse retinas (4 and 12 weeks old), we observed the formation of cell clusters located in the sub-retinal space (Fig. 4A). Integration of grafted cells into the remaining ONL, which consists solely of 1 or 2 nuclei thickness in 12 weeks old mice, was nevertheless not observed three weeks post-transplantation (Fig. 4A). Irrespective of that, the majority of donor photoreceptors had formed longitudinal, rhoEGFP–positive OS-like structures next to their DsRed-positive cell bodies (Fig. 4A′) as observed upon transplantation into healthy animals. We could not observe a consistent OS polarity, neither towards the RPE nor the ONL, of sub-retinal located donor cells in wt as well as in p347s animals. Electron-microscopic analysis revealed the formation of OSs with well-aligned disc membranes (in 8 out of 16 examined sub-retinal OSs), demonstrating the potential of transplanted PPCs to form native OS structures in a heavily degenerated retina, and this without cellular integration into the host tissues (Fig. 4A″, A′″).

To evaluate if such sub-retinal located donor photoreceptors are light-sensitive we analyzed the sub-cellular localization of transducin, a molecule of the light transduction pathway that is present in the OS in the dark and translocates to the inner segment/cell body upon light stimulation. Indeed immuno-histochemical analysis showed that transducin is predominantly present in OSs of donor photoreceptors in dark-adapted mice (Fig. 4C–C′″) whereas in light-adapted mice, it could only be detected in the cell body (Fig. 4B–B′″). Furthermore, as rhodopsin represents the first protein in the light transduction pathway and has to be highly expressed in functional photoreceptors, we analyzed the generation of rhodopsin by immunohistochemistry to determine its expression at the protein level. Indeed, labeled donor photoreceptors showed similar fluorescence intensities as endogenous photoreceptors in all transplantation paradigms (Fig. S1).

### The sub-retinal space provides a supporting environment for outer segment generation of transplanted photoreceptor precursor cells

**Figure 5:**
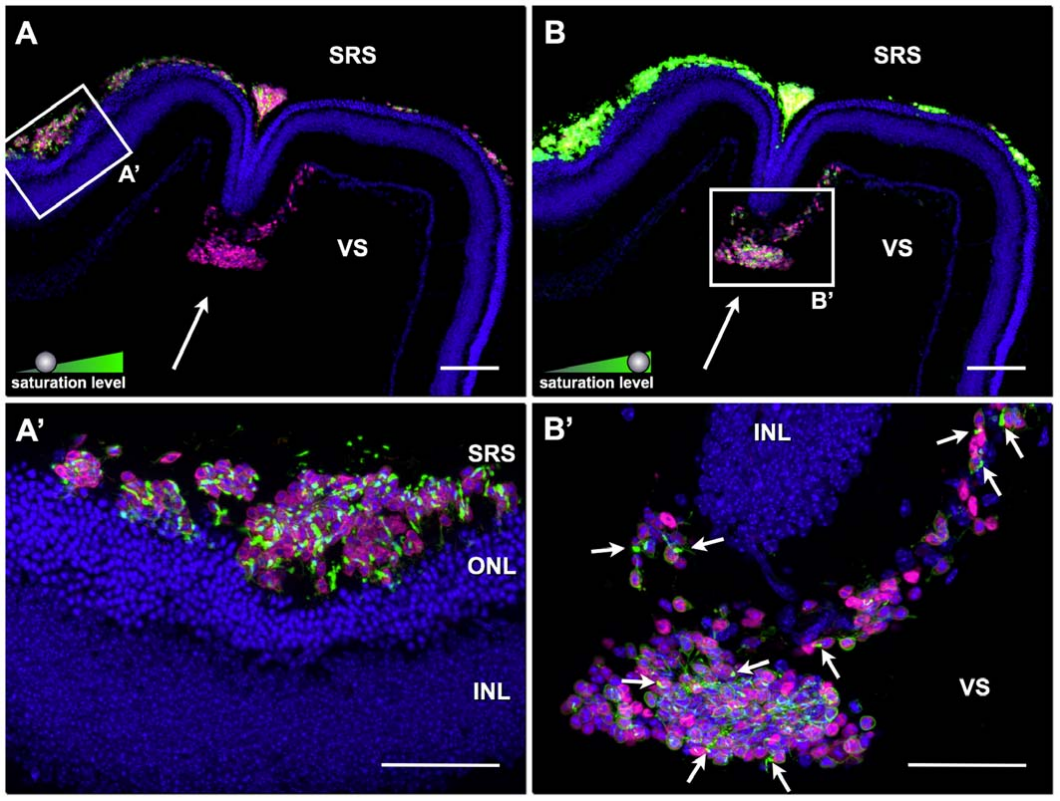
Differences in outer segment formation between sub-retinal and vitreal located transplanted photoreceptor precursor cells. CD73-enriched photoreceptor precursor cells (PPC) isolated from double-transgenic (rhoEGFP, actinDsRed) reporter animals were simultaneously transplanted into the sub-retinal (SRS) and vitreous (VS) space of 4-weeks old P347S hosts (A; B is the same image as A but with increased green fluorescence channel saturation level). Cell nuclei are highlighted with DAPI staining (blue). Donor cells (magenta) were detected in the SRS (e.g. boxed area in A) and VS (A and B, arrow) showing survival of transplanted cells in both locations. Whereas sub-retinally injected PPCs showed strong EGFP expression (green; e.g. boxed area in A), vitreally injected donor cells appeared almost devoid of EGFP signal (A, arrow). However, by increasing the saturation level of the green fluorescence signal channel in image A, faint EGFP signal could be detected in the vitreally located donor cells (B; B′ is a separate image with adjusted shutter time showing a magnification of the boxed area in B) demonstrating that donor cells located in the SRS had higher rhoEGFP expression levels than those in the VS (compare different cell populations in each individual panel A and B). Moreover, almost every single sub-retinal located donor PPC showed rhoEGFP labeled OSs (A′), in contrast to those located in the VS that generated only in few cases OS-like protrusions (B′, arrows). ONL, outer nuclear layer; INL, inner nuclear layer. Scale bars: 50 µm.

The data presented above demonstrate, that PPCs located to the sub-retinal space of both, wt and degenerated retinas, develop native-like OSs. This prompted us to investigate whether the OS formation by transplanted donor cells is a cell autonomous property or depends on environmental factors. To address this issue, we injected simultaneously donor PPCs into the sub-retinal space and the vitreous side of four-weeks old P347S transgenic hosts (Fig. 5).

**Figure 6:**
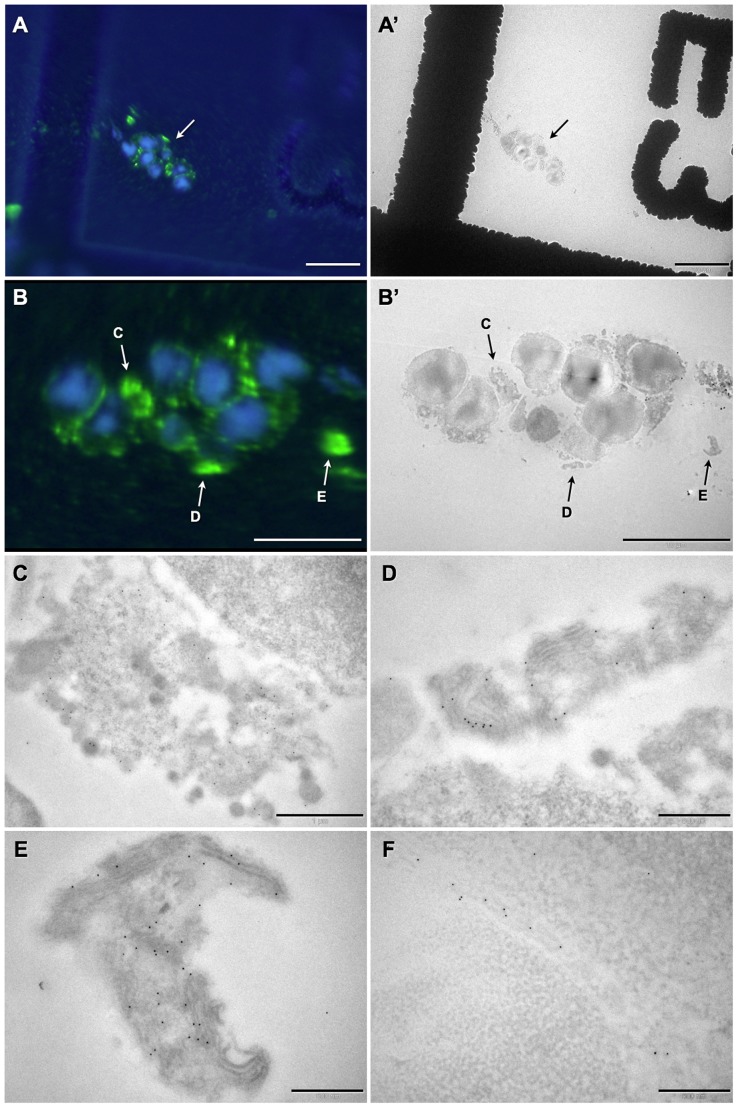
Vitreal-located transplanted photoreceptor precursor cells develop disturbed outer segment-like structures with misaligned disc membranes. CLEM analysis on resin sections allowed examination of vitreally-transplanted rhoEGFP–positive photoreceptor precursor cell aggregates. Cell nuclei are highlighted by DAPI staining (blue). A representative example is shown with fluorescence light microscopic- (A) and transmission electron microscopic-illumination (A′). The donor cells in A and A′ are shown at higher magnification in the respective panels B and B′. RhoEGFP-enriched structures (A, B; green, arrows) next to the cell bodies suggest the formation of OSs, and three of these (C, D and E) are displayed in the corresponding panels at a high-resolution level by TEM-analysis. These structures appear either as rhoEGFP-enriched cell debris (C) or rhoEGFP–positive OS-like structures with an abnormal organization/integrity including misaligned disc membranes (D, E). Additionally, mislocation of rhoEGFP to the plasma membrane of the soma was observed (F). Scale bars: A, A′: 20 µm, B, B′: 10 µm, C: 1 µm, D–F: 500 nm.

As shown above, PPCs located in the sub-retinal space developed the typical OS-like structures with high-levels of rhoEGFP expression (Fig. 5A, A′). In sharp contrast, when transplanted to the vitreous side, donor cells expressed significant lower amounts of rhoEGFP (Fig. 5A). Indeed, EGFP-positivity of vitreal located donor PPCs could only be visualized by increasing the saturation level of the green fluorescence channel of the same image (Fig. 5B; B′ is a higher magnification of the boxed area in B). Furthermore, FLM analyses suggested that in comparison to PPCs injected to the sub-retinal space (Fig. 5A′), the ones located on the vitreous side generated OS-like structures less frequently (Fig. 5B′, arrows; ultra-structurally only 7 out of 16 analyzed donor cells showed an OS-like structure) and that these were poorly developed (Fig. 5B′; see also Fig. 6A, B). CLEM analysis revealed that these structures are, in most cases (7 out of 7), altered OSs with abnormal arrangement including disorganized disc membranes (Fig. 6D, E). Occasionally, we observed a mislocalization of rhoEGFP to the plasma membrane of the soma (Fig. 6F). Taken together, these findings indicate that the sub-retinal space, in contrast to the vitreous one, supports the maturation of transplanted PPCs and proper biogenesis of OSs.

## Discussion

In the present study, we report a unique system that combines the use of transgenic mouse lines and CLEM to analyze the peculiar morphological details of the OS structure of transplanted PPCs. Three major cell biological observations were made using such biotechnical tools. First, we demonstrated that integrated PPCs develop into mature photoreceptor cells with appropriate OS structures. Second, we show that PPCs remaining in the sub-retinal space of wild-type as well as degenerated retinas generate also proper OSs and third, we revealed that environmental factors found in the sub-retinal space influence PPC differentiation and maturation.

PPCs have been studied as a possible therapeutic option for the replacement of lost photoreceptors in pre-clinical mouse models since their first successful transplantation already two decades ago [1], [3], [23], [24]. Recent reports have revealed their ability to integrate and differentiate into mature photoreceptors when transplanted into host retinas [10], [11], [13]. Indeed, studies indicated that upon integration into the host ONL donor PPCs develop adequate axonal terminals containing ribbon synapses located in the OPL whereas their ISs and OSs lie in the photoreceptor layer just above the outer limiting membrane [10]–[13]. Direct evidence for proper OS formation was however impeded due to the lack of adequate transgenic animal models allowing the accurate tracking of transplanted cells [10], [11], [13], [15]. For instance, most of the studies used soluble, cytoplasmic fluorescent reporter proteins, which in essence strongly limits their targeting towards the photoreceptor OS. Addressing the latter issue is particularly important given that the key feature of photoreceptors enabling correct light detection relies on the aligned disc membrane staples of their OS.

To settle such technical difficulty we used here transgenic animals as a source of donor cells where prospective OSs of transplanted cells could be visualized with a fluorescent reporter protein specifically targeted to the OSs by means of rhodopsin as a carrier protein. By combining CLEM with indirect immuno-labeling of the reporter-fusion rhodopsin-EGFP protein we demonstrated that integrated donor PPCs develop OSs with proper morphological structures that included the formation of a connecting cilium followed by aligned disc membrane staples. Moreover, short membrane evaginations growing from the connecting cilium at the base of OSs are detectable, consistent with the normal biogenesis of these photoreceptive structures. In addition, our investigations reveal unexpectedly that transplanted PPCs, which are not integrated into the host ONL, but remained in the sub-retinal space, generated also proper OSs. This observation is independent from the degenerative state of the host retina since a similar phenomenon was detected when PPCs were transplanted into severely degenerated retinas of P347S transgenic mice. Therefore, it appears that the formation of normal OSs from transplanted PPCs is independent of their integration into the ONL.

The cell biological properties of transplanted PPCs to form proper OSs independently of their integration within the host ONL may rely either on cell autonomous cues or cellular factors provided by the microenvironment found in the host retinas. To further dissect these possibilities we have differentially transplanted the PPCs into the sub-retinal space and vitreous side. Interestingly, we found that PPCs transplanted into the vitreous side express significant lower amounts of rhodopsin-EGFP and develop aberrant OSs by comparison to those injected to the sub-retinal space. Such data suggest that the maturation of PPCs as monitored by the expression of rhodopsin and formation of OS are provided by factors found specifically in the sub-retinal space. One major cellular player that could explain such singularity is the retinal pigment epithelium (RPE) adjacent to the sub-retinal space. Indeed, RPE cells have been shown to promote photoreceptor cell development, survival, neurite outgrowth and differentiation [25]–[27] as well as OS development, maintenance and regeneration [28], [29]. A direct connection of donor OSs with endogenous RPE cells does not seem to be necessary since OS formation is observed throughout the sub-retinal PPC population even when forming multiple layers of cells. As a consequence, we propose that factors secreted from the RPE have an important influence on the stimulation of transplanted PPCs to mature and form OSs. However, a correct alignment and growth towards the RPE was not observed. We therefore conclude, that these factors stimulate maturation and formation of OSs but are not sufficient for their correct alignment. Since in eye development the outgrowing OSs are tightly packed next to each other, it might be important for future studies to provide a scaffold for correct alignment and targeting of OSs developed by sub-retinal located PPCs.

The findings of this study are of prime importance to further develop this promising treatment strategy towards clinical application. Indeed, photoreceptor cell-based replacement therapy is founded on three main pillars. First, it is indispensable to establish a reliable and continuous source for transplantable materials. Second, a transplantation method providing a significant number of integration events and long-term survival of donor cells is a technical pre-requisite, and third, maturation including OS formation and functional integration of donor PPCs into the neural circuitry of the host retina are a must. Several teams are currently investigating the potential of diverse *in vitro* expandable cell populations for the generation of transplantable photoreceptors. These cell sources include i) expandable cells isolated from the retina, the so-called “retinal stem cells” [8], [9], [30], [31] and ii) pluripotent stem cells, i.e. embryonic stem cells (ESC) [4], [32] and induced pluripotent (iPS) stem cells [5], [6], [33]. Whether such produced or manufactured donor photoreceptors have however the potential to generate proper OS structures following grafting as demonstrated here for PPCs remains to be evaluated. Obviously, the double-(actinDsRed, rhoEGFP) transgenic animals might be used as cellular sources to generate such *in vitro* expandable donor cells to solve such issues.

Finally, we have documented that PPCs transplanted to the sub-retinal space have the potential to form proper photoreceptor OSs even when they are not integrated into the host ONL. A phenomenon observed as well in RD models (e.g. P347S: this study; rhodopsin knock-out: our unpublished observations). However, the lack of integration into the latter models raised some biomedical questions. Barber and colleagues have also linked a negative correlation between grade of degeneration and integrative potential into the host ONL in most retinal degeneration models studied [20], [21]. Such issues are particularly important when thinking about patients at late stage RD, i.e. who are blind or close to blindness and show a strong, if not a total degenerated ONL. Although, it appears unlikely at first glance that transplanted PPCs will successfully integrate into degenerated retinas of such affected patients (this study and [20], [21]), a recent study demonstrated that following transplantation into a slow degeneration mouse model of RP (i.e. Gnat1^−/−^ mice), PPCs are able to integrate into the host ONL and functionally connect to the neural circuitry by forming active synapses with endogenous interneurons [13]. Together with our results demonstrating OS formation and light sensitivity of donor photoreceptors in strongly degenerated retinas without integration, such achievements indicate that visual improvement might also be possible in late stage retinal degenerations. However, detailed investigations of functional integration of donor cells into the host neural circuitry at the cellular and behavioral level still have to be performed to evaluate the potential of transplanted photoreceptors in the heavily degenerated retina. Collectively, these findings represent a major step on the way towards the development of a cell-based replacement therapy of diseases characterized by photoreceptor loss.

## Materials and Methods

### Ethics Statement

All animal experiments were carried out in strict accordance with European Union and German laws (Tierschutzgesetz) and adhered to the ARVO Statement for the Use of Animals in Ophthalmic and Vision Research. All animal experiments were approved by the animal ethics committee of the TU Dresden and the Landesdirektion Dresden (approval number: 24D-9168.11-1/2008-33).

### Animals

Donor cells were isolated from retinas of PN4 transgenic reporter mice. The first transgenic animal is characterized by the expression of EGFP fused to human rhodopsin, which is introduced into the mouse rhodopsin locus (rhoEGFP mice) [16]. This reporter fusion protein facilitates the selective labeling and tracking of OS development since rhodopsin is specifically located therein. The second reporter mouse line was generated by crossing the rhoEGFP mouse line with the actin-DsRed one, in which DsRed expression [34] is driven by an ubiquitous active chicken beta-actin promoter (DsRed, B6.Cg-Tg(CAG-DsRed*MST)1Nagy/J from The Jackson Laboratory, Maine, USA; [35]). This double reporter mouse line allows the tracking of transplanted PPCs simultaneously to the development of their OSs in host retinas. Donor cells were either transplanted into wild-type mice (C57BL/6J; age: 2–4 months; n = 8) or heterozygous P347S mice (age: 4 and 12 weeks; n = 5), a model for autosomal-dominant RP [22]. For light-sensitivity studies, PN4, CD73-positive sorted actinDsRed, rhoEGFP donor cells were transplanted into p347s mice (n = 4), which were light-adapted (10 h in normal ambient light) or dark-adapted (24 h in complete darkness) 4 weeks after transplantation. All animal experiments were carried out in strict accordance with European Union and German laws (Tierschutzgesetz) and adhered to the ARVO Statement for the Use of Animals in Ophthalmic and Vision Research.

### Magnetic-activated cell sorting (MACS)

PPCs were enriched from 4-days old reporter mouse retinas using CD73 as a cell surface marker and immuno-paramagnetic separation methods as recently reported [12]. In brief, isolated retinas were dissociated, pelleted and dissolved in MACS buffer (Miltenyi Biotec, Bergisch Gladbach, Germany). After incubation with rat anti-CD73 antibodies (BD Pharmingen, Heidelberg, Germany), cells were washed and incubated with secondary anti-rat antibodies coupled with magnetic beads (Miltenyi Biotec), followed by a second washing step and filtering through a 30-µm pre-separation filter. Afterwards, magnetic separation was performed according to the manufacturer's protocol (Miltenyi Biotec). The CD73–positive cell fraction was collected and used for transplantations.

### Immuno-histochemistry

Whole mouse eyes were extracted from animals and processed as described before [12]. Briefly, after fixation in 4% paraformaldehyde (PFA), the retina was dissected out and sectioned into 30 µm thick slices using a vibrating microtome (model 1200; Leica, Wetzlar, Germany). For immuno-stainings, slices were incubated in blocking solution (1 h) before applying primary antibodies against transducin (rabbit anti-G_α t1_, Santa Cruz Biotechnology, Santa Cruz, USA) or rhodopsin (mouse anti-Ret-P1, Sigma-Aldrich, Munich, Germany) over night. Cy5-conjugated goat anti-rabbit or anti-mouse secondary antibodies (Jackson Immunoresearch, West Grove, USA) were used to visualize primary antibodies. To visualize cell nuclei, samples were stained with 4′,6-diamidino-2-phenylindole (DAPI; 1∶10,000; Sigma-Aldrich, Munich, Germany). Retinal sections were mounted on glass slides with AquaPolymount (Polysciences, Inc., Warrington, PA, US) for image acquisition.

### Correlative light and electron microscopy (CLEM)

Retina pieces were fixed with 4% PFA in 0.1 M phosphate buffer (PB, pH 7.4) overnight at 4°C. Fixed samples were processed for PLT-embedding in K4M as described [17], [18]. In brief, samples were dehydrated in a graded series of ethanol at progressively lower temperatures, infiltrated in mixtures of ethanol/K4M at −35°C and embedded in resin. Polymerization occurred via UV-irradiation at −35°C. Ultra-thin sections were used for immunofluorescence and/or immuno-EM [17]–[19], [36]. Sections were blocked with 1% BSA in PBS, incubated with rabbit anti-GFP (TP 401 from Torrey Pines or ab 290 from Abcam) followed by either Alexa488 (or Alexa555) conjugated secondary antibodies or by protein A 10-nm gold, and finally counterstained with DAPI (1 µg/ml). For CLEM of the very same section, ultra-thin sections were mounted on nickel finder grids (200 mesh, EMS, # G 200F1-Ni) and stained with anti-GFP antibody and protein A gold. After post-fixation with 1% glutaraldehyde/PBS, the sections were incubated with goat anti-rabbit Alexa488 and counterstained with DAPI. Labeled EM-grids were mounted between two coverslips in 50% glycerin/water [36] and analyzed at the fluorescence microscope, then de-mounted, washed several times in water and stained with aqueous uranyl acetate before EM inspection.

### Transplantation

For transplantation of cell suspensions into the sub-retinal or vitreal space, adult (i.e. 2–4 months old) wild-type (C57BL/6J) or heterozygous P347S (4 or 12 weeks old) mice were anesthetized by an intra-peritoneal injection of medetomidine hydrochloride (Dormitor®, Pfizer, Berlin, Germany; 0.01 mg/10 g body weight) and ketamine (Ratiopharm, Ulm, Germany; 0.75 mg/10 g body weight), and fixed in a head holder. Pupils were dilated by drops of 1% tropicamid (Mydrum®, Dr. Mann Pharma GmbH, Berlin, Germany) and 2.5% phenylephrine (TU-Dresden pharmacy). A Hamilton syringe with a blunt 34-gauge needle was inserted tangentially through the conjunctiva and sclera and placed under visual control into the sub-retinal space, i.e. between the retina and RPE producing a bullous retinal detachment, or into the vitreal space, i.e. in front of the ganglion cell layer. 1 µl of cell suspension containing approximately 400,000 cells was injected per eye. For recovery experimental animals received an injection of atipamozole hydrochloride (Antisedan®, Pfizer, 0.1 mg/10 g body weight) for reversal of medetomidine.

### Fluorescence and TEM image acquisition and data analysis

Light microscopical images were taken using a Z1-Imager fluorescence microscope with Apotome and AxioCam Mrm camera (Zeiss, Germany). Before EM image acquisition, the fluorescence of CLEM samples was imaged using a Keyence BZ 8000 (Keyence, Neu-Isenburg, Germany). EM sections were analyzed using a Philips Morgagni 268 (FEI; Philips, Amsterdam, Netherlands) at 80 kV and images were taken with the MegaView III digital camera (Olympus, Hamburg, Germany). Images were analyzed and prepared for publication using ImageJ, AxioVision (Zeiss), GNU Image Manipulation Program (Gimp) and iWork Keynote (Apple).

## Supporting Information

Figure S1 **Rhodopsin expression in rhoEGFP-positive OSs.** Rhodopsin expression was detected in rhoEGFP-positive OSs of integrated donor cells in wt (A–A′″) as well as in rhoEGFP-positive OSs of sub-retinal located cells in P347S mice (B–B′″). DsRed-positive, transplanted PPCs (A, magenta) show following integration into the host wt ONL typical green fluorescent, rhoEGFP-positive OSs at the tip of their ISs (A, A′, white and A′″, green) highlighted by arrows in A–A′″. Rhodopsin staining (A″, white and A′″, magenta) reveals a relatively equal expression throughout the OSs of the wt host. The rhoEGFP-positive OSs co-localize with rhodopsin immuno-staining (compare A′ and A″, arrows), indicated by white overlap staining in A′″ (arrows). Following transplantation into P347S mice sub-retinal located DsRed-positive PPCs (B, magenta) develop the characteristic rhoEGFP-positive OSs adjacent to the cell body (B, B′, white and B′″, green). The rhoEGFP fluorescence co-localizes with rhodopsin staining (compare B′ and B″, arrows), indicated by white overlap staining in B′″. Similar rhodopsin expression levels are detectable in host and donor cells (A, B) suggesting a native expression of rhodopsin in rhoEGFP-positive OSs of transplanted and integrated as well as sub-retinal located PPCs. Scale bars: 10 µm.
(TIF)
