# Supplementary figures and images for: Outer Segment Formation of Transplanted Photoreceptor Precursor Cells
Source: PLoS One. 2012 Sep 28;7(9):e46305. doi: 10.1371/journal.pone.0046305 (PMC3460822; doi:10.1371/journal.pone.0046305)

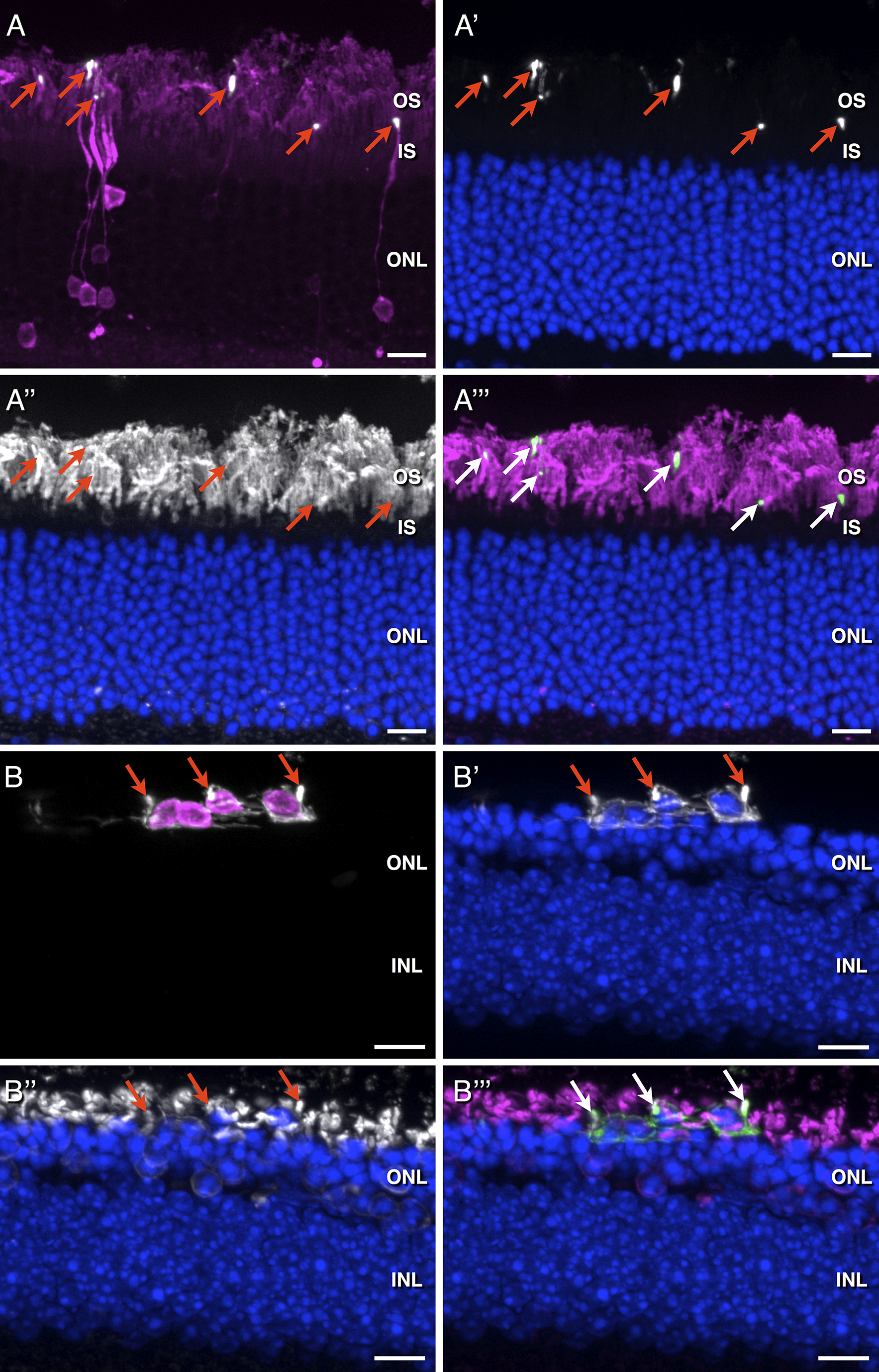

Supplement: Figure S1 — Rhodopsin expression in rhoEGFP-positive OSs. Rhodopsin expression was detected in rhoEGFP-positive OSs of integrated donor cells in wt (A–A′″) as well as in rhoEGFP-positive OSs of sub-retinal located cells in P347S mice (B–B′″). DsRed-positive, transplanted PPCs (A, magenta) show following integration into the host wt ONL typical green fluorescent, rhoEGFP-positive OSs at the tip of their ISs (A, A′, white and A′″, green) highlighted by arrows in A–A′″. Rhodopsin staining (A″, white and A′″, magenta) reveals a relatively equal expression throughout the OSs of the wt host. The rhoEGFP-positive OSs co-localize with rhodopsin immuno-staining (compare A′ and A″, arrows), indicated by white overlap staining in A′″ (arrows). Following transplantation into P347S mice sub-retinal located DsRed-positive PPCs (B, magenta) develop the characteristic rhoEGFP-positive OSs adjacent to the cell body (B, B′, white and B′″, green). The rhoEGFP fluorescence co-localizes with rhodopsin staining (compare B′ and B″, arrows), indicated by white overlap staining in B′″. Similar rhodopsin expression levels are detectable in host and donor cells (A, B) suggesting a native expression of rhodopsin in rhoEGFP-positive OSs of transplanted and integrated as well as sub-retinal located PPCs. Scale bars: 10 µm. (TIF) [file pone.0046305.s001.tif]
